# Insights into the Effects of Pore Size Distribution on the Flowing Behavior of Carbonate Rocks: Linking a Nano-Based Enhanced Oil Recovery Method to Rock Typing

**DOI:** 10.3390/nano10050972

**Published:** 2020-05-18

**Authors:** Amin Rezaei, Hadi Abdollahi, Zeinab Derikvand, Abdolhossein Hemmati-Sarapardeh, Amir Mosavi, Narjes Nabipour

**Affiliations:** 1 Abdal Industrial Projects Management Co. (MAPSA), Tehran 1456914477, Iran; arezaei@parspetro.com (A.R.); Z.derikvand@mapsaeng.com (Z.D.); 2 Department of Petroleum Engineering, Science and Research Branch, Azad University, Tehran 1477893855, Iran; habdollahi@srbiau.ac.ir; 3 Department of Petroleum Engineering, Shahid Bahonar University of Kerman, Kerman 7616913439, Iran; hemmati@uk.ac.ir; 4 College of Construction Engineering, Jilin University, Changchun 130600, China; 5 Faculty of Civil Engineering, Technische Universität Dresden, 01069 Dresden, Germany; 6 Kalman Kando Faculty of Electrical Engineering, Obuda University, 1034 Budapest, Hungary; 7 Department of Mathematics, J. Selye University, 94501 Komarno, Slovakia; 8 Institute of Research and Development, Duy Tan University, Da Nang 550000, Vietnam; narjesnabipour@duytan.edu.vn

**Keywords:** nanomaterials, pore throat size distribution, mercury injection capillary pressure, interfacial tension, contact angle, enhanced oil recovery, surfactant, nanoparticle

## Abstract

As a fixed reservoir rock property, pore throat size distribution (PSD) is known to affect the distribution of reservoir fluid saturation strongly. This study aims to investigate the relations between the PSD and the oil–water relative permeabilities of reservoir rock with a focus on the efficiency of surfactant–nanofluid flooding as an enhanced oil recovery (EOR) technique. For this purpose, mercury injection capillary pressure (MICP) tests were conducted on two core plugs with similar rock types (in respect to their flow zone index (FZI) values), which were selected among more than 20 core plugs, to examine the effectiveness of a surfactant–nanoparticle EOR method for reducing the amount of oil left behind after secondary core flooding experiments. Thus, interfacial tension (IFT) and contact angle measurements were carried out to determine the optimum concentrations of an anionic surfactant and silica nanoparticles (NPs) for core flooding experiments. Results of relative permeability tests showed that the PSDs could significantly affect the endpoints of the relative permeability curves, and a large amount of unswept oil could be recovered by flooding a mixture of the alpha olefin sulfonate (AOS) surfactant + silica NPs as an EOR solution. Results of core flooding tests indicated that the injection of AOS + NPs solution in tertiary mode could increase the post-water flooding oil recovery by up to 2.5% and 8.6% for the carbonate core plugs with homogeneous and heterogeneous PSDs, respectively.

## 1. Introduction

Residual oil remains in the reservoirs after conventional water flooding is often regarded as the target for enhanced oil recovery (EOR) processes. A comprehensive understanding and evaluation of the displacement efficiency represent a fundamental requirement for oil production forecast and field development planning [1,2,3]. At a laboratory scale, similar rock types have different residual oil saturations after water flooding, and a particular relation between the macroscopic characteristics and the flow behavior of the porous medium is yet to be developed [4,5]. Wardlaw et al. presented a low-cost method for estimating the amount of residual oil in a carbonate reservoir based on the observational evaluation of rock sections and pore casts [6]. They also stated that the oil recovery increases with the pore-to-throat diameter ratio, although this hypothesis is yet to be tested experimentally. Magara placed emphasis on the significant contribution of pore size to the oil production efficiency [7]. Chatzis et al. studied the effect of pore size on the residual oil saturation and ended up finding no significant association between the residual oil saturation and the rock pore size, although they observed an increase in the amount of the trapped oil at higher aspect ratios [8]. Elgaghah et al. considered the influence of pore size distribution (PSD) on the displacement efficiency in oil-bearing reservoirs [9]. They employed the image processing technique to find the pore size distribution of the rock samples in order to design the optimum microbial EOR process regarding the PSD of the porous media.

The amount of oil remained in the reservoir after the conventional water flooding was found to be dependent on the porosity and permeability of the porous medium [10], both of which are known to be influenced by the PSD of the reservoir rock [11]. Hence, it is essential to study the effect of PSD on the flow behavior of the reservoir rock to understand the governing mechanisms of the fluid flow through the porous medium. Al-Shalabi and Ghosh studied the effect of permeability contrast on the efficiency of water flooding in different porous media consisting of a glass micromodel (representing a high-permeability medium) and a set of sandstone core samples (representing a low- to medium-permeability medium) [1]. They employed three techniques to measure the water displacement efficiency, namely linear core flooding, micro model flooding, and imbibition by ultracentrifugation. Interestingly, their results showed a higher ultimate oil recovery in the lower-permeability medium. Gharibshahi et al. investigated the effects of pore shape, pore connectivity, pore heterogeneity, and tortuosity on the enhanced oil recovery (EOR) and the breakthrough time of micro models of nanofluid flow using computational fluid dynamics (CFD) [12]. Based on their results, they argued that a random distribution model tended to resemble the fluid flow in a hydrocarbon reservoir through the trapping effect on the flowing behavior that could not be investigated by such a model. However, to the best of our knowledge, there are only limited research works considering the effect of nanoscale confinement on the flow behavior of the fluid phase for water flooding in carbonate rocks. So far, there have been many studies elaborating on the effects of pore geometry on the permeability in the geological literature [13,14], but most of the relevant works have been focused on the single-phase fluid flow [15]. To better clarify the effects of PSD on the flow behavior of a reservoir rock, it is important to collect samples of similar rock types. Conventional reservoir rock typing has been defined as the classification of the reservoir rock based on its petrophysical properties acquired through wireline logs, porosity-permeability correlations, mercury injection curves, and geological features [5,16,17,18,19,20]. As a basis for reservoir rock classification, the pore geometry can be assessed based on the capillary pressure, which, in turn, can be better measured by the mercury injection analysis rather than other methods [21]. Most of the aforementioned research works have pointed out the relationship between the PSD and the residual oil saturation based on the analyses performed on a synthetic structure (i.e., micro model or sand-packs), making their results unreliable for using in actual reservoir studies. Accordingly, the present research is an attempt to fill in the research gap of investigating the effects of the PSD on the flow behavior of a porous medium.

Surfactant solution injection has been recommended as an efficient method to improve the oil displacement efficiency, provided the injection amount is sufficient, particularly for depleted reservoirs [22]. The surfactants have been widely studied as additives to decrease the oil–water interfacial tension (IFT) and residual oil saturation [23,24,25,26,27,28,29,30]. They also act as a high-grade agent to alter the rock surface wettability [31,32]. The ability of surfactant solutions to alter the wettability of solid surfaces depends on the properties of both the surfactant and the neighboring reservoir rock [33]. The primary effect of the surfactants that contributes to enhanced oil recovery is the reduction of the oil-brine IFT, even at low concentrations [26,27]. Adsorption of surfactants on the rock surface, on the other hand, reduces the efficiency of the EOR process [30,34]. So, in the present study, attempts were made to minimize the surfactant loss upon the adsorption on the rock surface.

Additionally, in recent years, several scholars have studied the feasibility of applying nanotechnology to different aspects of the petroleum industry [35,36,37,38,39,40,41]. Reservoir engineers have implemented nanoparticles (NPs) for different purposes in the petroleum industry, including the EOR. Surfactant-silica NPs solutions have drawn attention for their application in the EOR process, where they can help decrease the surfactant loss, reduce the NP dosage, increase the particle stability, and enhance the efficiency [42,43]. Roustaei obtained an additional 10.0% original oil in place (% OOIP) by imbibition of CTAB solution in 6.8 wt.% saline water at ambient conditions [44]. By integrating TX-100 (nonionic surfactant) with silica NPs, Zhao et al. could enhance the oil recovery by 8.0% of OOIP in the presence of 3.0 wt.% NaCl at 80 °C [45]. Ogolo et al. stated that some NPs are suitable for EOR projects, and the presence of ethanol could efficiently improve the EOR performance. Besides, they showed that the mechanisms by which NPs could improve the oil recovery included reduction of oil–water interfacial tension, oil viscosity, and mobility ratio, and modification of the rock surface wettability and the porous medium permeability [46]. Rezaei et al. studied combinations of various surfactants (namely, cocamidopropyl betaine (CAPB), sodium dodecylbenzene sulfonate (SDBS), coconut di-ethanol amide (CDEA), and linear alkylbenzene sulfonic acid (LASA)), alkalis (i.e., sodium carbonate and sodium tetraborate), and nanoparticles (viz., silica, zinc oxide, and cloisite 30B) for EOR from dolomite rock sample [47]. They could achieve almost 19.7% and 12.2% enhancement in ultimate oil recovery from dolomite rock samples by CAPB+sodium carbonate and CAPB+silica nanoparticle, respectively. Suleimanov et al. could achieve up to 18% more oil recovery, in comparison with the surfactant flooding, by combining NPs with the surfactant solution. They attributed this oil recovery improvement to the decrease in the interfacial tension of the nanoparticle-containing system [48]. Handraningat et al. studied the effect of NPs concentration on the efficiency of the nano-EOR flooding and could reduce the probability of pore blockage of sandstone rocks by applying an optimum range for the concentration of NPs [49]. Despite the widespread use of nanotechnology in the EOR schemes, there is still little knowledge on the application of the surfactant–NP composites as a new trend in the field of chemical EOR.

In this study, firstly, the unsteady-state oil–water relative permeability curves of two core plugs with similar flow zone indices (FZI) but different PSDs were compared during synthetic formation water (SFW) flooding, as a secondary EOR technique. Afterward, an extended series of experiments, including the IFT tests between the aqueous phases containing the alpha olefin sulfonate (AOS) surfactant + silica NPs and the oil phase, and the contact angle measurements, were carried out to determine a promising mixture of the surfactant and the NPs for EOR purposes. Zeta potential measurements and dynamic light scattering (DLS) analysis were also performed to evaluate the stability of the silica NPs in the presence of the anionic surfactant. Finally, as a first-time investigation into the efficiency of the surfactant + NP flooding considering the effect of PSDs, core flooding experiments were done on core plugs of the same rock type but different pore throat size distributions.

## 2. Materials and Methods 

In the following section, materials used in this study and their information are listed. An introduction to experimental procedures and the setups used for tests are also covered in this segment.

### 2.1. Materials

#### 2.1.1. Surfactant

**Figure 1:**
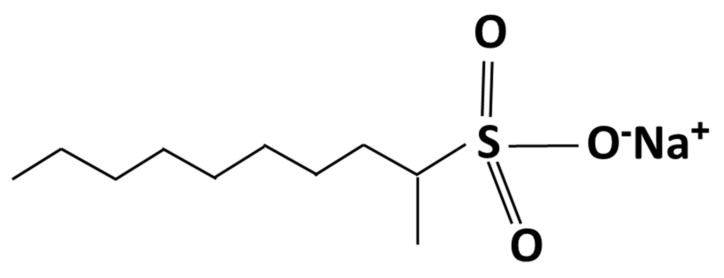
Chemical structure of alfa olefin sulfonate.

Alpha olefin sulfonate (AOS) in solid white powders is an anionic surfactant that was used in this study. Figure 1 presents the chemical structure of the AOS surfactant.

#### 2.1.2. Nanoparticle

**Figure 2:**
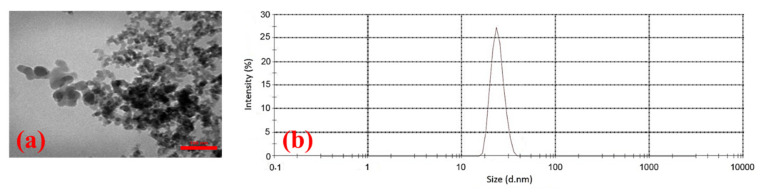
(**a**) TEM image and (**b**) DLS analysis of the nanoparticle.

Hydrophilic silica nanoparticles (non-porous, 25 nm, the specific surface area of 200 m^2^/g, the density of 2.4 g/cm^3^, and purity +99.5%), which were purchased from Sigma-Aldrich Company, Taufkirchen, Germany were used in experiments. Transmission electron microscopy (TEM) analysis on the silica powders was performed using a transmission electron microscope (Model: Philips EM208S 100KV, Nicosia, Cyprus). In order to investigate the size distribution of silica NPs, a dynamic light scattering (DLS) test was performed using the Malven ZS Nano analyzer (Malven Instrument Inc., London, UK). Results of TEM and DLS analysis were depicted in Figure 2a,b, respectively. As the results of the DLS test revealed, the size distribution of silica NPs is 18 nm to 38 nm, with an average size of 25 nm. According to the thin pore throats of the carbonate rocks, determination of the maximum radius of flocculated NPs is a crucial matter in NPs flooding. As determined by the DLS analysis, the maximum flocculated size of silica NPs is almost 38 nm, which confirms the ability of NPs to pass through the pore throats (minimum size of 0.05 µm to 2 µm as measured by mercury injection capillary pressure (MICP) tests).

#### 2.1.3. Synthetic Brine

A solution of 180 g of sodium chloride (supplied by Merck, Darmstadt, Germany) in one liter of deionized water (DIW) was used as the synthetic brine. The equivalent amount of NaCl is determined for making synthetic brine with consideration of equal ionic strengths for the synthetic and the formation brine.

#### 2.1.4. Crude Oil

**Figure 3:**
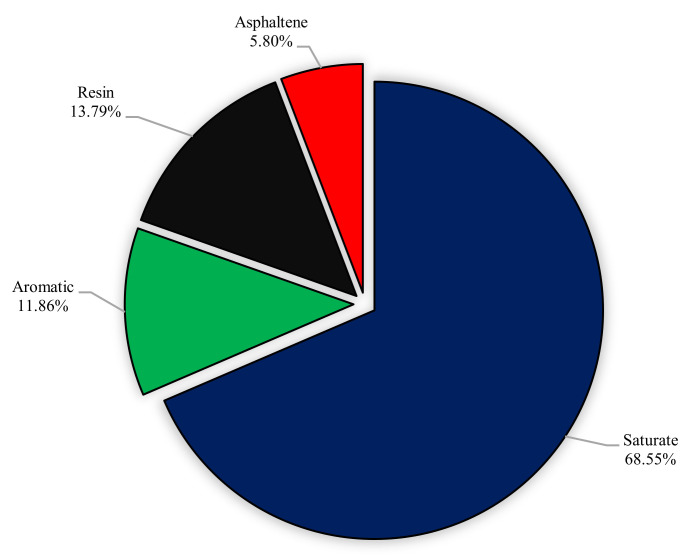
The results of SARA analysis for used crude oil.

**Figure 4:**
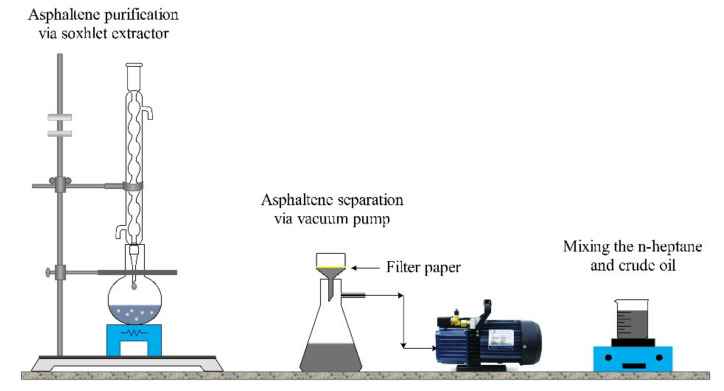
Schematic of the asphaltene extraction process.

The crude oil obtained from one of the Iranian oil fields with the viscosity of 15 cp and the gravity of 31.5° API was used for aging processes and experimental sections. SARA analysis was performed for better characterization of studied crude oil. Separation of saturates, aromatics, resins, and asphaltenes was performed as follows: initially, asphaltene fraction of the crude oil was extracted according to the IP-143 procedure described in detail elsewhere [50]. Briefly, 1 g of the crude oil was mixed with 30 mL of n-heptane, and refluxed for 60 min and placed in a dark room for about 12 h. Asphaltenes was filtrated by filter paper and washed by n-heptane until the complete removal of impurities. Subsequently, the filter paper was washed by toluene. Finally, solvents were evaporated, and asphaltenes were obtained. The soluble fraction of crude oil in n-heptane was further separated to saturate, aromatic, and resin by silica gel packed column chromatography. Saturate, aromatic, and resin fractions were separated by elution of n-hexane, toluene, and toluene/methanol (90/10), respectively. Figure 3 and Figure 4 show the results of SARA analysis and a schematic view of the asphaltene extraction method, respectively.

#### 2.1.5. Rock Samples

**Figure 5:**
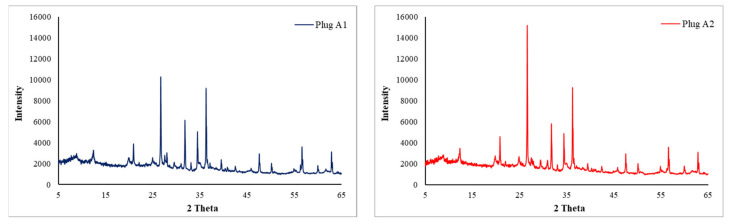
Results of XRD analysis on the powdered A1 (left) and A2 (right) rock samples.

Carbonate rock samples used for core flooding experiments were selected from one of the Iranian oil reservoirs. Results of X-ray diffraction (XRD) analysis on the powdered rock samples are depicted in Figure 5.

Routine specifications of the core plugs are listed in Table 1.

### 2.2. Experimental Procedures

#### 2.2.1. Rock Typing

In order to select representative rock samples between more than 20 core plugs, the common, inexpensive, and efficient flow zone index (FZI) method was applied, and two core plugs with similar rock types were picked. FZI is a practical and well-known method in reservoir engineering to simply identify rock types [51,52,53]. Results and the method for FZI calculations are described in Section 3.1.

#### 2.2.2. Zeta Potential Measurement

To inquire about the effect of AOS surfactant on the stability of NPs suspensions, two solutions (a) 0.01 wt.% of silica NPs and (b) 0.01 wt.% of AOS + 0.01 wt.% of silica NPs, both in DIW were prepared, and the zeta potential for the two solutions was measured. It is noteworthy that the reported zeta potential values for each solution are the average of three times measurements.

#### 2.2.3. IFT Measurements

A series of IFT measurements were performed to find the optimum concentrations for AOS surfactant and silica NPs in the EOR solution. Drop shape analyzer (DSA 100, KRÜSS, Hamburg, Germany) apparatus was applied in both IFT measurements and determination of the oil droplets contact angle on the oil-wet rock sections. For the purpose of IFT determination between the oil and the aqueous phases, oil droplets were injected into the aqueous phase (as the bulk phase, contains surfactant and NPs) via a syringe; and the image of the oil droplet as it was about to be apart was used for IFT determinations. In-house software was applied for calculating the value of IFTs.

#### 2.2.4. Contact Angle Measurements

**Figure 6:**
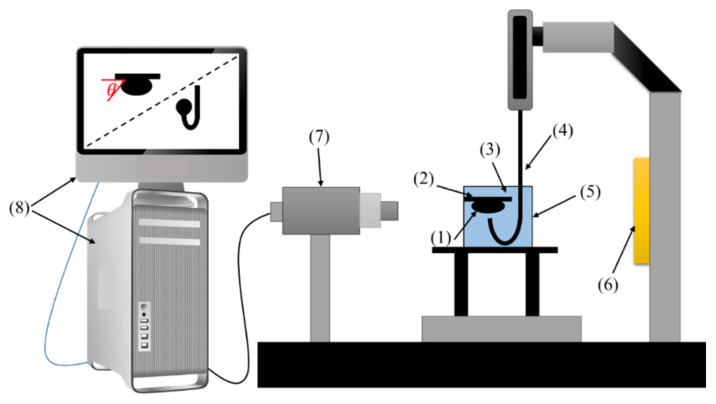
Schematic of the setup for interfacial tension IFT) and contact angle tests: (1) oil droplet, (2) rock section, (3) aqueous solution, (4) needle, (5) cell, (6) light source, (7) CCD camera, (8) processor.

Contact angles of the sessile oil droplets on the rock surface were measured via DSA 100 apparatus. In all experiments, the aqueous phase composed of different concentrations of AOS + SiO_2_ NPs in SFW was used as the bulk fluid. Figure 6 demonstrates a schematic of the equipment used for IFT and contact angle measurements.

#### 2.2.5. MICP Tests

**Figure 7:**
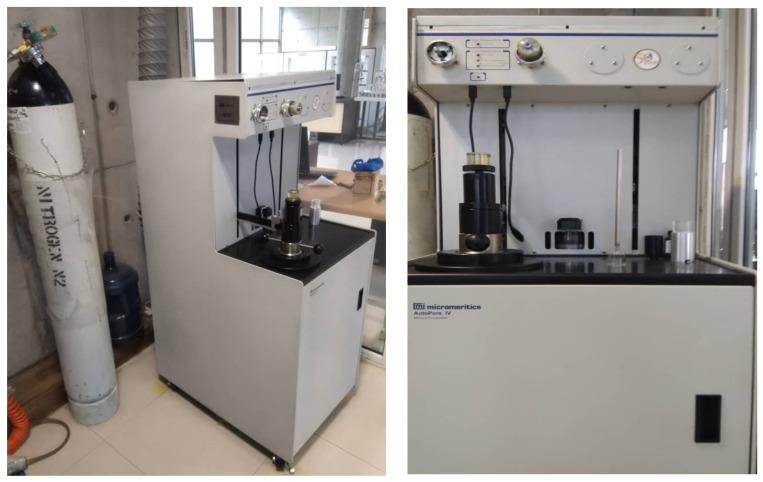
Setup used for mercury injection capillary pressure (MICP) tests.

The distribution of pressure-volume relationships can be explored through mercury injection and monitoring the entry of mercury into a pore system. As the mercury is the non-wetting phase, it should overcome the capillary forces to go into the pore volumes, and the intrusion process will only progress after applying an ever-increasing pressure. A Micromeritics Autopore IV 9500 Porosimeter apparatus, GA, USA (see Figure 7) was used for MICP measurements.

Following is the procedure of MICP tests applied in this study to determining PSDs in core cuttings:

- Weighting the cleaned and dried sample.
- Selecting a proper penetrometer according to the pore volume of the core cutting.
- Weighting the penetrometer containing the cutting.
- Loading the penetrometer to the low-pressure chamber.
- Measuring the bulk volume of the cutting.
- Increasing the pressure of mercury injection from 0.5 to 30.0 psig incrementally and monitoring the amount of mercury intrusion at each pressure step.
- Loading the sample into the high-pressure chamber.
- Injecting the mercury into the core cutting and raising the pressure (up to 60000 psia) incrementally.
- After equilibrium is established in the last step, the injection pressure is reduced to atmospheric pressure.
- Mercury saturations are calculated as a percentage of the pore volume at each pressure, and the pore volume used for calculation of mercury saturation obtained from the maximum intrusion volume.

#### 2.2.6. Core Flood Experiments

**Figure 8:**
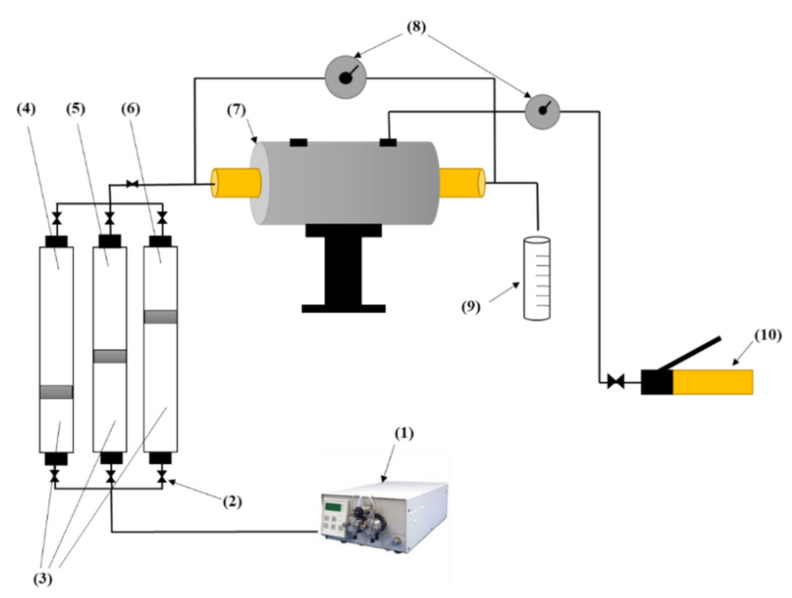
Diagram of the setup used for core flooding tests: (1) high-pressure liquid chromatographic (HPLC) pump, (2) valve, (3) distilled water, (4) synthetic formation water, (5) dead oil, (6) surfactant/nanoparticle solution, (7) core holder, (8) differential pressure transmitter, (9) calibrated burette, (10) overburden pressure.

Applicability of the surfactant–NPs solution as an EOR method was examined through displacement tests, and the efficiency of this EOR method in different PSDs was investigated. The core plug is placed into a hassler-type stainless steel core holder, and overburden pressure about 1000 Psi was applied using a manual confining pump. The liquid accumulator and EOR solution cylinder connected to the core holder and a precise pressure transmitter (Model: Rosemount 3051CD, MN, USA) was installed to measure the differential pressure between the inlet and the outlet of the core sample. The HPLC pump injects the distilled water to the transfer vessels and pushes the piston up, which causes the fluids (i.e., SFW, surfactant–nanoparticle solutions, or the crude oil) to be injected into the core plug by a specific rate. The outlet pressure in all core flooding experiments was ambient pressure. Eventually, produced fluid was collected in a calibrated burette. Figure 8 depicts a diagram of the setup used for core flooding experiments.

## 3. Results and Discussion

### 3.1. Core Sample Selection

FZI rock typing technique was applied on more than 20 core plugs cached from the depths of 3604 m to 3610 m of the reservoir, and the two core plugs which were principally alike were selected. This method includes the three following equations (see Equations (1)–(3)) [51]:

RQI = 0.0314 √ (k⁄*φ*), (1)

*φ_z_* = *φ*/(1 − *φ*), (2)

FZI = RQI⁄*φ_z_*, (3)

where RQI is the rock quality index (μm), *K* is the permeability of the core plug (mD), *φ* is the effective porosity (fraction), *φ_z_* is the normalized amount of the effective porosity, and the ratio of RQI to normalized porosity is called the flow zone index (μm).

The calculated FZI for cores A1 and A2 were 0.52 and 0.56, respectively, which indicates that the two samples have similar FZI; thus, they are classified into the same rock type or hydraulic flow unit (HFU) [51,54,55]. 

### 3.2. Static Stability of the Solution

**Figure 9:**
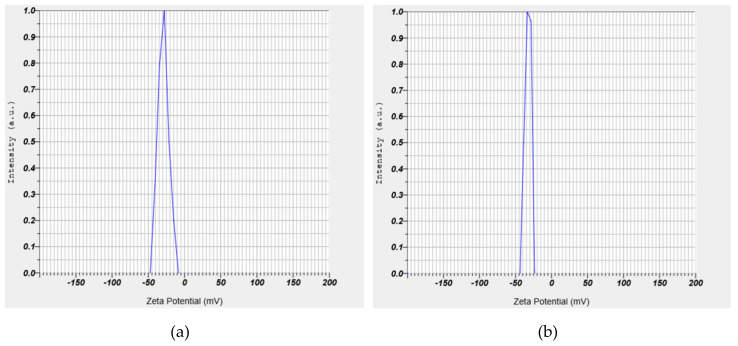
Zeta potential measurements for (**a**) SiO_2_ and (**b**) alpha olefin sulfonate (AOS) + SiO_2_ solutions.

**Figure 10:**
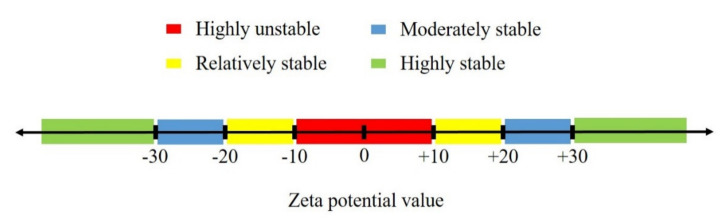
Different ranges of zeta potential values and the level of stability of nanoparticles into the aqueous phase.

In order to investigate the stability of NPs into the aqueous solutions, zeta potential measurements were conducted on two pre-prepared suspensions, including (a) 0.01 wt.% of SiO_2_ and (b) 0.01 wt.% of SiO_2_ + 0.01 wt.% of AOS surfactant, in DIW. Results of zeta potential measurements for the NPs and the AOS–NPs are shown in Figure 9. A negative zeta potential value signifies that the forces between the silica NPs and the AOS surfactant monomers used in this study are repulsive, which causes the nanoparticles not to be accumulated [56]. Investigating colloid stability is one of the most common applications of zeta potential data. Figure 10 shows guidelines classifying NPs-dispersions with different ranges of zeta potential values [56,57].

The average amounts of zeta potential measurements were reported −29.8 and −32.5 for SiO_2_ and AOS + SiO_2_ solutions, respectively. As can be deduced from Figure 10, the results of zeta potential determinations depicted that the presence of the anionic surfactant could efficiently improve the stability of silica NPs into the solution. 

In order to investigate the size distribution of the SiO_2_ NPs, separately and in combination with the AOS surfactant, two aqueous solutions containing (a) SiO_2_ (0.005 wt.%) and (b) AOS (0.005 wt.%) + SiO_2_ (0.005 wt.%) were prepared and the DLS analysis was performed on both. The results of DLS tests demonstrated that the radius of aggregated NPs as 178 and 146 nm for the solutions (a) and (b), respectively. The smaller radius of the aggregated NPs for the AOS + SiO_2_ solution indicates the lower inclination of the NPs to sedimentation.

### 3.3. IFT between the Crude Oil and Aqueous Solutions

**Figure 11:**
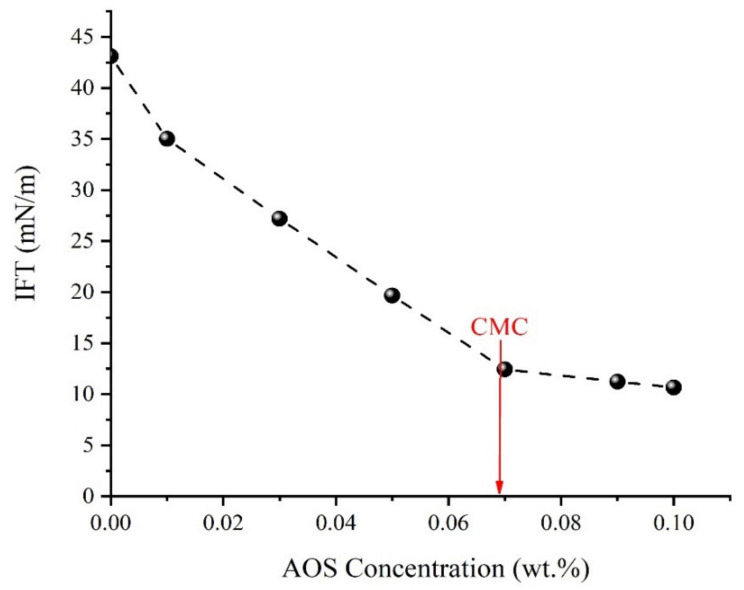
Crude oil–aqueous solution IFT at different concentrations of AOS surfactant.

Two series of IFT measurements were conducted to finally determine the optimum concentrations for the AOS surfactant and the silica NPs in aqueous solution. The first series of oil–water IFT measurements were performed, and the critical micelle concentration (CMC) for the AOS surfactant was measured as 0.067 wt.%. Figure 11 shows the results of IFT measurements between different concentrations of the AOS surfactant in SFW and crude oil.

**Figure 12:**
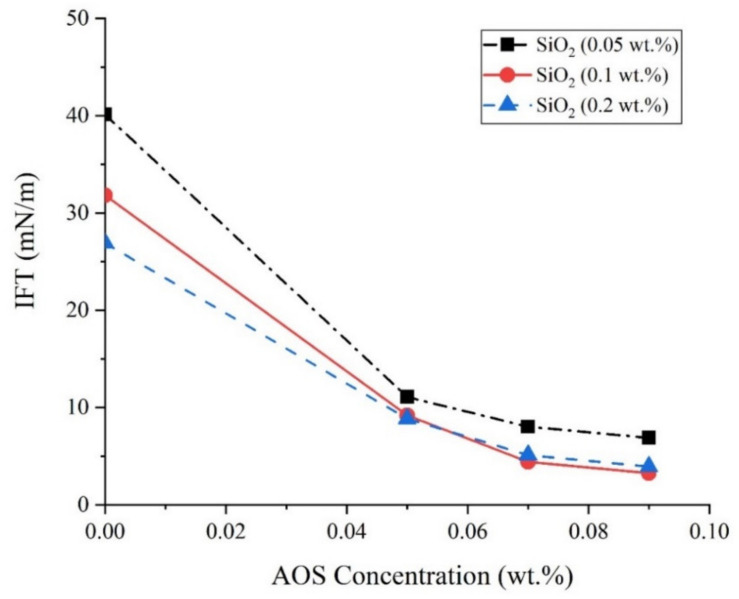
IFT values between the crude oil and the aqueous solution.

In the next series of experiments, constant concentrations of 0.06, 0.07, and 0.08 wt.% of AOS surfactant and 0.05, 0.1, and 0.2 wt.% of SiO_2_ NPs were added to SFW, and the oil–aqueous phase IFT values were determined. As can be concluded from Figure 12, the addition of silica NPs to the surfactant solution causes a further reduction in IFT values. In these concentrations for silica NPs, the optimum concentration for the presence of NPs in the aqueous solution was determined as 0.1 wt.%.

### 3.4. Wettability Measurement

**Figure 13:**
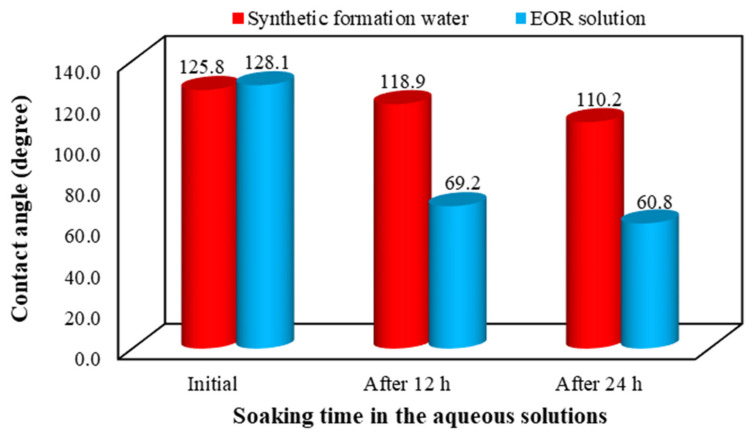
Effects of soaking time of the rock sections in red: synthetic formation water and blue: the enhanced oil recovery (EOR) solution on the oil-rock contact angle.

In this section, we measured the variation of the contact angle of oil droplets on oil-wet rock sections with the aid of a sessile drop technique. For this purpose, the restored rock sections were used to measure the wettability alterations by soaking them into the SFW and the EOR solution. The contact angle of oil droplets on rock sections was determined (a) before aging, (b) after aging, (c), and (d) after 12 and 24 h soaking into the aqueous phases, respectively. Figure 13 shows the results of contact angle measurements at different times when the SFW and the EOR solution (0.067 wt.% of AOS + 0.1 wt.% SiO_2_) are the bulk phases.

**Figure 14:**
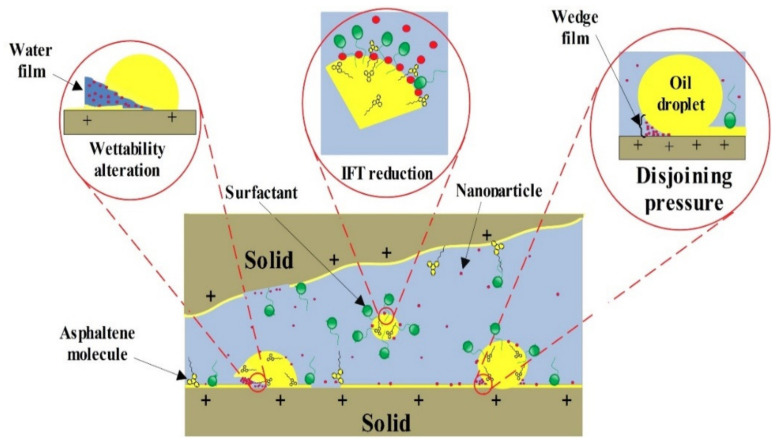
Schematic view of surface interactions of the carbonate rock–oil–aqueous phase (containing surfactant + NPs) system.

The disjoining pressure defined as the pressure required to overcome the fluid’s adhesion force to the solid surface to remove the liquid from the surface. In this case, the disjoining pressure is defined as the attractive interactions between the aqueous phase and the oil film. Through this mechanism, a wedge film of the discontinuous oil phase attached to the rock surface is created by the NPs, which were dispersed into the aqueous phase (see Figure 14). As a specific point of view, the formation of these wedge films, which are considerably influenced by Brownian motion and electrostatic repulsive forces between nanoparticles, is the primary mechanism for wettability alteration of the rock sections from oil-wet to water-wetness [58]. On the other hand, by increasing the concentration of nanoparticles, the repulsive forces between the NPs, and also the intensification of these repulsive forces due to the presence of surfactant monomers with the same electrical charge, increases the disjoining pressure and Brownian motion. So, the performance of the aqueous phase in decreasing the contact angle values improves as the concentration of nanoparticles increases [58,59,60]. However, the point to note here is that increasing the concentration of NPs, in addition to raising the costs, enhances the possibility of NPs deposition, which leads to formation damage due to plugging the rock pores and pore throats [61,62].

**Figure 15:**
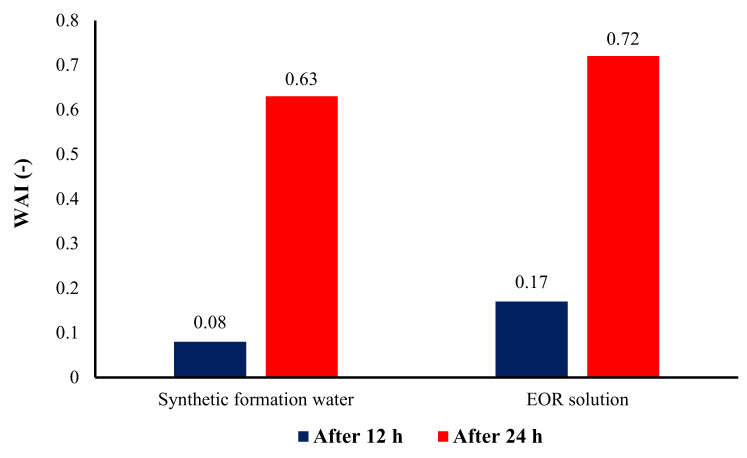
Effects of soaking time in different aqueous solutions on the wettability alteration index (WAI) of the carbonate rock sections.

To have a better comparison of the ability of different aqueous solutions in altering the rock surface wettability, the effect of the initial value of the contact angle was eliminated. Thus, the wettability alteration index (WAI) was determined using Equation (4) [63], and the results are shown in Figure 15.

WAI = (*θ_0_* − *θ_f_*)/(*θ_0_* − *θ_i_*), (4)

where *θ_0_* and *θ_i_* are contact angles after and before aging, respectively, and *θ_i_* is contact angle after being in contact with the EOR solution. As deduced from Equation (4); the closer the value of WAI to 1, the more the wettability changes due to the aqueous bulk solution. Figure 15 also illustrates the superiority of the EOR solution performance in changing the wettability of rock surfaces towards water-wetness in comparison with the SFW.

### 3.5. Determination of the PSD

The fraction of the pore volume injected against pore throat radius is represented through the data of MICP tests. As shown in Equation (5), the differential of pore volume injected provides a function for pore throat size distribution.

PSD = dv/dlog(r),(5)

The differential equation will be calculated numerically. The central difference method (Equation (6)) is used to calculate PSD as:

PSD_i_ = (V_i+1_ – V_i–1_)/(log(r_i+1_) − log(r_i–1_)), (6)

Using Equation (7), the PSDs of the core plugs were smoothed and then normalized to 1 by applying the Equation (8):

PSD_i_ = (PSD_i–1_ + 2PSD_i_ + PSD_i+1_)/4, (7)

PSD_normal,i_ = PSD_i_/PSD_max_, (8)

**Figure 16:**
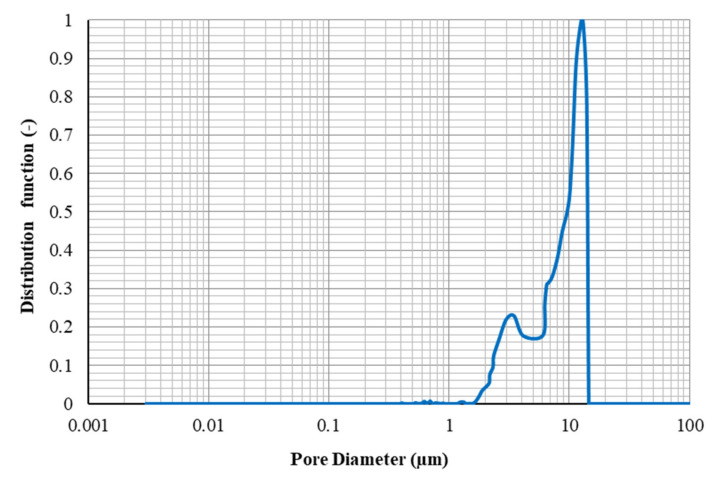
Pore throat size distribution for core A1.

**Figure 17:**
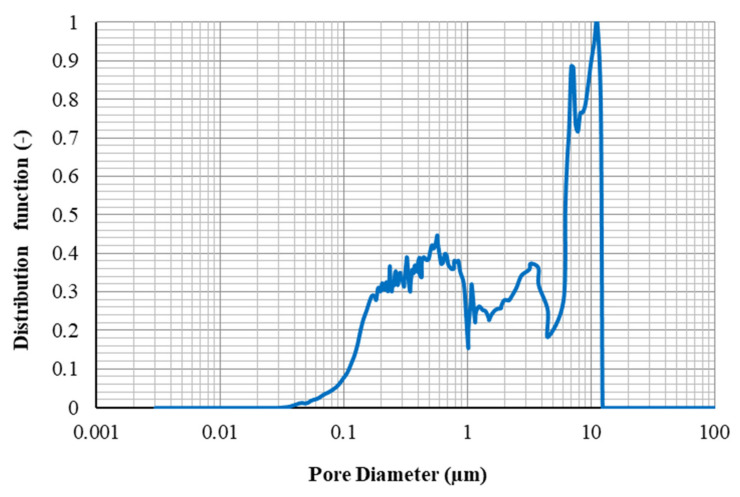
Pore throat size distribution for core A2.

The normalized PSD is represented in graphical shape along with the saturation of mercury against pore throat radius. Figure 16 and Figure 17 show the PSDs determined through MICP tests for the core plugs A1 and A2, respectively.

As can be seen from Figure 17, the PSDs for core A2 cover a broad range of pore throat sizes from 0.03 to 14 µm uniformly, and the average diameter of the pores is 763.9 nm. Although the range of pore throat diameter for sample A1 (from 1.8 to 15 µm- see Figure 16) is comparable to that for sample A2, the average pore diameters for this rock sample (7609.5 nm) is far greater than that for sample A1. Therefore, this can be concluded that for core sample A1, there are larger pore throat sizes in it, which may not be connected effectively, and core sample A2, pores would have better connections together because of the extensive range of pore throat radiuses.

The total pore areas for the core samples A1 and A2 were also estimated as 0.273 and 0.035 (m^2^/g), respectively.

### 3.6. Flooding Experiments

**Figure 18:**
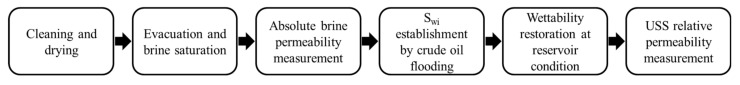
Steps to core sample preparation.

In order to investigate the effect of pore throat size distributions on the flowing behavior of the core samples, two series of flooding experiments were conducted on each core plug. Figure 18 depicts the core preparation procedure carried out before unsteady-state relative permeability measurements.

Core plugs were immersed in crude oil at 90 °C and 3500 psi for 21 days to restore the wettability of core plugs to reservoir conditions.

In the interest of investigating the effect of different pore throat size distributions on the flowing behavior of the oil and water phase, two-phase unsteady-state relative permeability curves were established based on the data of oil production and pressure drop across the core plug during SFW flooding.

**Figure 19:**
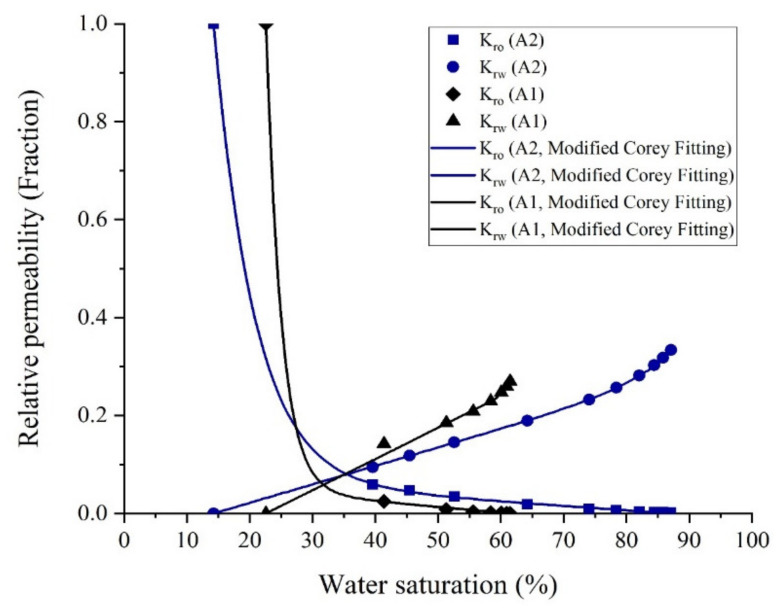
Relative permeability curve for cores A1 and A2.

After loading the core plug into the core holder, crude oil was injected into the sample to (1) replace the oil into the core plug with fresh oil and (2) to obtain oil permeability at irreducible water saturation (*S_wi_*). Figure 19 indicates the relative permeability curves for core samples A1 and A2. As depicted in Figure 19, the amount of oil recovered by water flooding increases as the homogeneity of the pore throat size and pore connectivity increase. The cross point of water and oil relative permeability curves for core plugs A1 and A2 were at water saturation of 32% and 36%, respectively. It meant that, although the aging process could not sufficiently change the wettability of rock samples to oil wetness, it was more efficient in core sample A2, because of the better connections between pores.

The residual oil saturation for the core sample A2 is about 25% less than the core A1. Unlike what Wardlaw and Magara [6,7] stated theoretically, the higher oil recovery for a rock sample obtained from core flooding experiments could not exclusively be the result of having a higher absolute gas permeability or larger pore sizes, and the PSDs and the homogeneity of pore sizes are of particular importance.

**Figure 20:**
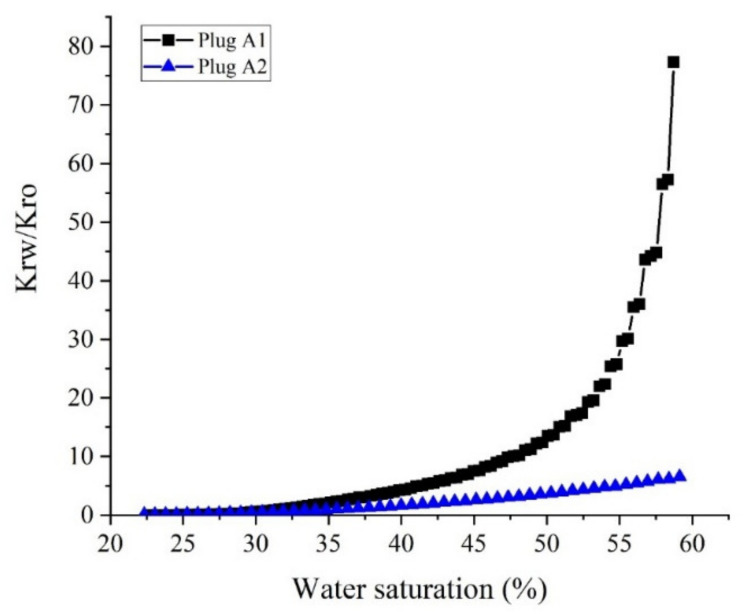
The ratio of water to oil relative permeability (*K_rw_/K_ro_*) for core samples A1 and A2 at overlapped water saturations.

To compare the ability of the pore network of the core samples in oil transmission, the relative permeability ratio of water and oil (*K_rw_/K_ro_*) in a similar range of water saturation (*S_w_*) for core sample A1 and A2 are represented in Figure 20. As demonstrated in Figure 20, the ratio of water to oil relative permeability data for core sample A2 is much lower than that for core sample A1, which implies the higher ability of the core sample A2 in oil transmissibility than that for core sample A1. Furthermore, it can be concluded from Figure 20 that in similar aging conditions, the core A1 showed more oil-wet behavior, which this behavior can be attributed to its large pore size distribution and more homogeneous structure.

The experimental procedure for measuring the PSD and the USS relative permeability are available in Supplementary Materials (see Section S.1 in Supplementary file and Figures S1 to S3 and Tables S1 to S3).

### 3.7. Enhanced Oil Recovery Tests

**Figure 21:**
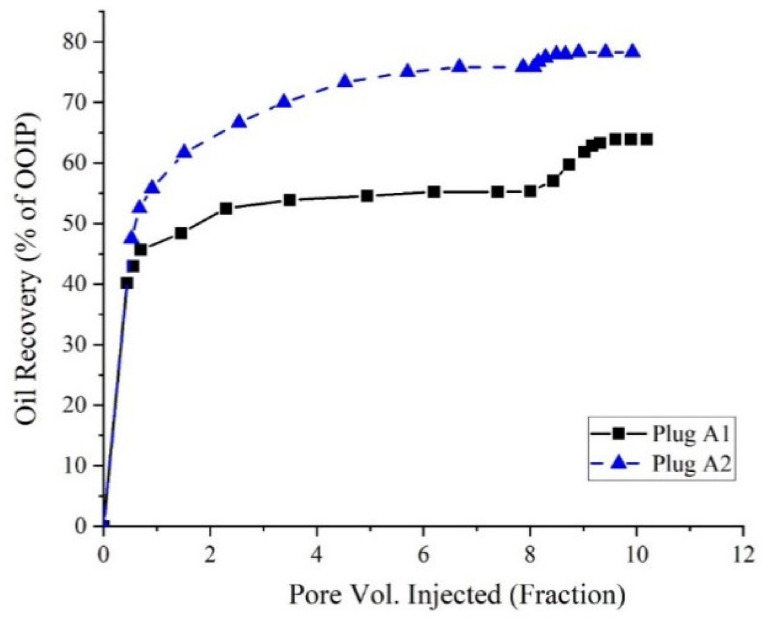
Oil recovered from the secondary and tertiary oil recovery processes.

After flooding with formation water, a solution of SFW containing 0.1 wt.% AOS surfactant + 0.1 wt.% silica NPs was flooded into the core samples with a constant flow rate of 0.02 cc/min. Figure 21 shows the amount of oil recovered after secondary and tertiary modes of displacement tests performed on the core plugs.

For sample A2, most of the oil is recovered through secondary water flooding. This is because of the homogeneity of the pore throat sizes in this core plug, which causes that the aqueous phase sweeps the oil easily out of the pores, and reaches the residual oil saturation after water flooding to a very low value. However, in sample A1, a large amount of oil remained unswept after flooding with SFW. Early water breakthrough time and also the high amount of unswept oil remained within the core plug after water flooding confirmed the presence of some large pore throats into the core plug, which drives the aqueous phase flow to the end of the plug.

Distribution of some large pores alongside a negligible range of small pores in the structure of sample A1 increases the probability of having a very high aspect ratio in the pore network of the core plug. Therefore, the significant amount of oil left behind into the core plug after secondary core flooding tests makes this rock sample an appropriate candidate for EOR plans. The best way to recover the unswept oil remaining in the core plug after water flooding is to alter the rock surface wettability towards water-wetness and reduce oil–water IFT; thus, oil transmissibility into the porous media will increase. NPs are able to move through the small and inaccessible pores and can alter the wettability of the surface of the pores to water-wetness. The addition of the AOS surfactant to the NPs suspension will increase the stability of the NPs into the aqueous phase and also reduces the IFT between the remained oil into the pores and the aqueous phase, and subsequently, the residual oil, which is remained unswept can be recovered. Application of surfactant + NPs as the EOR technique could enhance oil recovery from core plugs A1 and A2 up to about 2.5% and 8.6%, respectively.

## 4. Conclusions

In the present study, for the first time, the simultaneous effects of PSDs and a chemical-based EOR method were investigated experimentally through an extended series of experiments, and the following conclusions are obtained:

- The presence of 0.1 wt.% silica NPs in combination with the AOS surfactant (CMC), reduces the IFT value from 12.5 mN/m to 4.4 mN/m. The results of zeta potential and DLS tests confirmed the synergy of AOS surfactant and silica nanoparticles.
- Results of contact angle measurements, as well as oil–water relative permeability tests, indicate the capability of the AOS + SiO_2_ solution in altering the wettability of carbonate rocks toward water-wetness.
- For core sample A2 with a wide range of uniform pore sizes, the amount of oil recovered by secondary water flooding was more than that for core plug A1. Thus, in conventional water flooding, the homogeneity of the pore sizes is more important than the dimensions of the pores.
- Based upon the results of core flooding experiments, which showed more oil recovery in water flooding for the plug with homogeneous PSDs, it can be deduced that using the results of MICP tests is a more reliable method than the FZI technique for rock typing in carbonate rocks.
- At the same range of water saturation, the ratio of water to oil relative permeability for core plug with better pore connectivity is much lower than that for the core plug with poor pore connections. This shows how the better connection between the pores can influence oil recovery by water flooding, which is in good agreement with the results of core flooding tests.
- Results of displacement tests outlined that applying the surfactant + NPs solution as an EOR technique could recover up to 2.5% and 8.6% more oil from the core plugs with homogeneous and non-homogeneous PSDs, respectively.

## Figures and Tables

**Table 1:** The specifications of the core samples.

Sample	Depth (m)	Length (mm)	Diameter (mm)	Grain Density (g/cm^3^)	Porosity (%)	Gas Permeability (mD)	*S_wi_* (%)
A1	3609.42	48.12	37.43	2.70	15.73	1.51	22.58
A2	3606.39	47.50	37.51	2.70	13.48	1.05	14.23

## Supplementary Materials

The following are available online at https://www.mdpi.com/2079-4991/10/5/972/s1, Figure S1: Desiccator used for vacuum saturation of the core plugs, Figure S2: Schematic view of the setup used for USS water/oil relative permeability measurements, Figure S3: Water/oil relative permeability curves, Table S1: Routine core specifications and conditions of the water/oil relative permeability test, Table S2: The results of USS oil/water relative permeability test, Table S3: Parameters of the Modified Corey Model.

## Abbreviations

AOS	Alpha olefin sulfonate
DIW	Deionized water
DLS	Dynamic light scattering
EOR	Enhanced oil recovery
FZI	Flow zone index
IFT	Interfacial tension
MICP	Mercury injection capillary pressure
NP	Nanoparticle
PSD	Pore throat size distribution
SFW	Synthetic formation water
TEM	Transmission electron microscopy
WAI	Wettability alteration index
XRD	X-ray diffraction
