# Supplementary material for: Insights into the Effects of Pore Size Distribution on the Flowing Behavior of Carbonate Rocks: Linking a Nano-Based Enhanced Oil Recovery Method to Rock Typing
Source: Nanomaterials (Basel). 2020 May 18;10(5):972. doi: 10.3390/nano10050972 (PMC7712098; doi:10.3390/nano10050972)
Supplement: Supplementary file 1 [file nanomaterials-10-00972-s001.pdf]

nanomaterials

MDPI

Supplementary Material

Insights into the Effects of Pore Size Distribution on the Flowing Behavior of Carbonate Rocks: Linking a Nano-based Enhanced Oil Recovery Method to Rock Typing

Amin Rezaei [1], Hadi Abdollahi [2], Zeinab Derikvand [1], Abdolhossein Hemmati-Sarapardeh [3,4], Amir Mosavi [5,6,7]*, and Narjes Nabipour [8]

1 Abdal Industrial Projects Management Co. (MAPSA), Tehran 1456914477, Iran, arezaei@parspetro.com (A.R.); Z.derikvand@mapsaeng.com (Z.D.)
2 Department of Petroleum Engineering, Science and Research Branch, Azad University, Tehran 1477893855, Iran, habdollahi@srbiau.ac.ir
3 Department of Petroleum Engineering, Shahid Bahonar University of Kerman, Kerman 7616913439, Iran, hemmati@uk.ac.ir
4 College of Construction Engineering, Jilin University, Changchun 130600, China
5 Faculty of Civil Engineering, Technische Universität Dresden, 01069 Dresden, Germany,
6 Kalman Kando Faculty of Electrical Engineering, Obuda University, 1034 Budapest, Hungary
7 Department of Mathematics, J. Selye University, 94501 Komarno, Slovakia
8 Institute of Research and Development, Duy Tan University, Da Nang 550000, Vietnam, narjesnabipour@duytan.edu.vn
* Correspondence: amir.mosavi@weimar-uni.de

Received: 18 March 2020; Accepted: 8 May 2020; Published: 18 May 2020

S.1 Unsteady-State (USS) Oil/Water Relative Permeabilities Measurement

1. Introduction

A wide range of relative permeability measurements can be used to obtain engineering parameters for oil and gas field design purposes. Relative permeability is defined as the ratio of the effective permeability of each phase at a specific saturation to the base permeability of the rock. The base relative permeability could be water permeability at residual oil saturation (K_w @ S_{or}), oil permeability at irreducible water saturation (K_o @ S_{wi}), or absolute air permeability (K_g). In this study, K_o @ S_{wi} was employed as the base permeability for the relative permeability analysis because this parameter was measured after aging the core samples, just before the test in the same conditions. However, by selecting any of the aforementioned permeability values as the base permeability, the ratio of the relative permeability of the tested fluids would not change. Conventionally, relative permeability is determined in laboratories by Unsteady-State (USS), steady-state (SS), or Centrifuge methods.

In the present study, oil/water relative permeability experiments were performed using a core flooding apparatus (Hassler type uniaxial core holder and a Scientific System Inc., MAPSA Co., Tehran, Iran, constant rate dual piston HPLC pump, Bellefonte, PA, USA, for fluid injection) on selected samples in ambient conditions by the volumetric measurement USS method. The following is a summary of the procedure and the calculations applied to assess the oil/water relative permeability for a two-phase system.

2. The Procedure and Calculations used for Oil/Water Relative Permeability Measurements

We can classify the steps of the oil/water relative permeability measurements into two sections: first, the core sample preparation steps (from 1 to 4), and second, the relative permeability determination steps (from 5 to 12):

- After cleaning the core plugs (following the procedure which is mentioned in Section 2.1.4 of the paper), the core plugs were placed in an oven for about six hours at 60 °C to dry.
- An unsteady state gas permeameter and porosimeter (Coreval 30, Villeurbanne, France) were applied to determine the porosity and gas permeability of the core plugs.
- After that, the pore volume of the core samples were filled with reservoir brine in a vacuum saturation setup (see Figure S1).

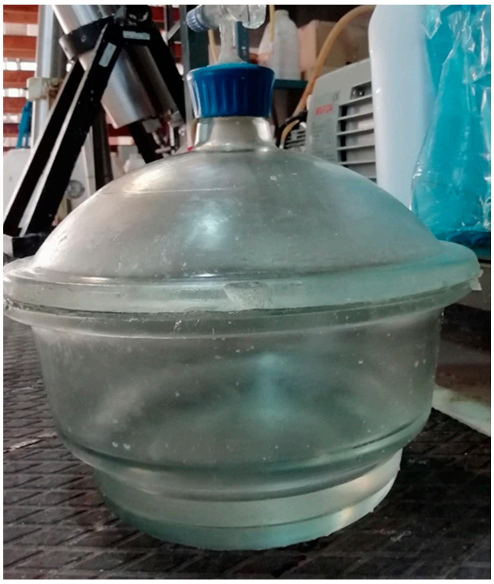

Figure S1. Desiccator used for vacuum saturation of the core plugs.

- After measuring brine permeability by core flooding tests, the crude oil was flooded into the plugs to reach irreducible water saturation. The plugs were then soaked into the oil and placed in an oven at 80 °C for almost 30 days to restore the wettability condition of the reservoir.
- Next, the plug was loaded into the Hassler type core holder, into which the crude oil was injected at a constant rate. After stable differential pressure (DP) was attained, the DP at four dissimilar flow rates was recorded and used to determine the effective oil permeability at irreducible water saturation (K_o @ S_{wi}). Later, such values were used as the base permeability for relative permeability calculations.
- At a constant injection rate, a calibrated burette and quality controlled pump were used to inject the displacing phase into the plug at a fixed flow rate.
- The aqueous solution began to be injected into the plug at a fixed rate, and the produced fluid volume, time, and pressure differences between the two sides of the plug were recorded regularly. Figure S2 shows a schematic view of the setup used for the relative permeability analysis.

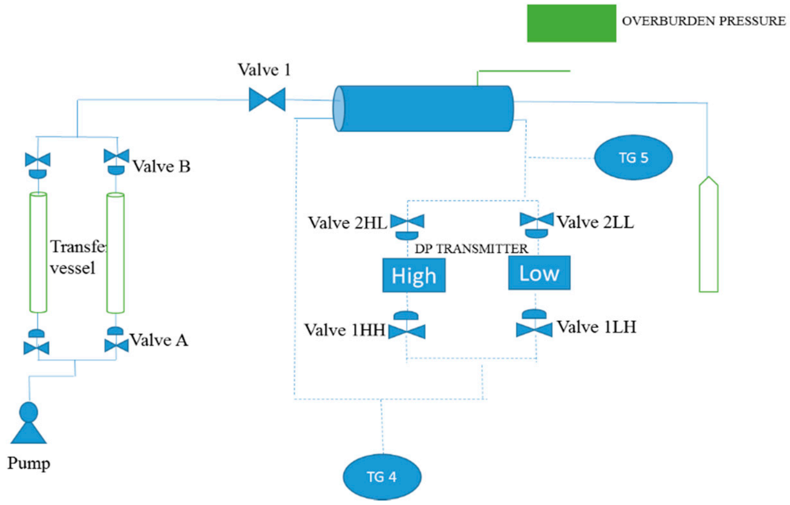

Figure S2. Schematic view of the setup used for USS water/oil relative permeability measurements.

- A fraction collector was used to measure the effluent fluid volume at determined times.
- Injection of the aqueous phase continued until no more oil was produced (i.e., the water cut reached 99.9%).
- For each of the collection tubes, and after leaving enough time for separation to occur, the water and oil volumes were recorded.
- The cumulative volume of each phase was calculated to be applied in plotting the oil recovery versus the pore volume of the injected brine.
- Over the course of the experiments, the injection and production rates, relative permeability values, and average water saturation ($S_w(avg)$) should be determined. The following is the equation used to determine the average saturation of the aqueous phase in each sample:

$$S_w(\text{avg}) = S_{wi} + \frac{V_o(\text{produced})}{\text{Pore volume}} \quad (1)$$

Toth et al. produced equations to estimate the relative permeabilities for USS fluid displacement experiments [1]. Applying these equations in various experimental data demonstrated the feasibility of this method in the calculation of oil/water relative permeability [2–4]. Accordingly, using this method, which is more precise and applicable than the technique employed by Johnson et al. (i.e., nonlinear regression) [5], it is necessary to sketch the following nonlinear relations: $S_w(avg)$ versus cumulative water injected (*wi*) and the pressure ratio (1/Ir) versus wi. Subsequently, is necessary to plot tangents so that curves meet the ordinates ($S_w(avg)$, indicating the mean S_w within the core.

Therefore, in the present study, the method used by Toth et al. was applied to assess the two-phase relative permeability curves. Details of this method are mentioned in their paper (see Reference [1] for more information). Moreover, it should be mentioned that most of the experimental data are noisy, and an efficient smoothing method is required for computations. The modified Corey or modified power-law models were used to fit the relative permeabilities data.

3. Example of USS Oil/Water Relative Permeability Experiments

In this section, an example of oil/water relative permeability tests conducted on a plug, of which the specifications and test conditions are listed in Table S1, is presented. All the calculations for determining the relative permeability were done as stated in the Toth et al. method.

Table S1. Routine core specifications and conditions of the water/oil relative permeability test.

System	USS oil/water imbibition	Initial water saturation (%)	24.79
Data processing	Toth et al. method	**Flow direction**	Horizontal
Core sample length (cm)	4.92	**Temperature (°C)**	Ambient
Core sample diameter (cm)	3.81	**Oil type**	Synthetic oil
Porosity (%)	13.93	**Oil viscosity 25°C**	1.47
Absolute gas permeability (mD)	4.33	**Water viscosity @ 25°C**	1.45
Absolute brine permeability (mD)	2.75	**Net confining pressure (psi)**	400
Effective oil permeability at S_{wi} (mD)	2.12	**Constant down pressure (cc/min)**	Ambient
Base permeability	$K_o@S_{wi}$	**Constant rate (cc/min)**	0.5
Saturated pore volume (cc)	7.18	**Test duration (min)**	120

USS relative permeability measurement was applied to measure the oil/water relative permeabilities. A summary of the results of the experiment is listed in Table S2. It is worth mentioning that as recommended by the Toth et al. method, the experimental data related to the period after the breakthrough time were analyzed.

Table S2. Results of the USS oil/water relative permeability test.

Time (min)	Differential press. (psi)	Injected water vol. (PV)	Produced water vol. (cc)	Produced oil vol. (cc)	S_w (avg.) (%)	S_w outlet (%)	Oil recovery (% of OOIP)	K_{rw} (Fraction)	K_{ro} (Fraction)	K_{rw}/K_{ro}
**0**	34.02	0	0	0	24.79	24.79	0	0	1	0
**9**	86.26	0.83	0.6	3.9	79.11	78.14	72.22	0.343	0.025	13.80
**11**	81.96	0.77	1.3	4.2	83.29	79.58	77.78	0.365	0.018	20.52
**15**	73.06	1.04	3.2	4.3	84.68	81.38	79.63	0.398	0.011	37.73
**20**	67.48	1.39	5.66	4.35	85.37	82.68	80.56	0.427	0.006	66.31
**33**	61.52	2.3	12.1	4.4	86.07	84.26	81.48	0.477	0.003	177.34
**53**	58.21	3.89	22.1	4.4	86.07	85.21	81.48	0.528	0.001	451.76
**73**	56.21	5.08	32.1	4.4	86.04	85.66	81.48	0.564	0.001	851.74

After measuring the water/oil relative permeabilities of the plug, the Modified Corey model was applied to fit an appropriate plot onto the experimental results. Table S3 illustrates the values for the variables of the model [6].

Table S3. Parameters of the Modified Corey Model.

Parameter	Water relative permeability (K_{rw})	Oil relative permeability (K_{ro})
Alpha	2.897	1.373
Y_{max}	0.541	1.000
H	0.000	0.000
V	3.974	2.195

The obtained data from the oil/water relative permeabilities test were plotted, and the results are shown in Figure S3.

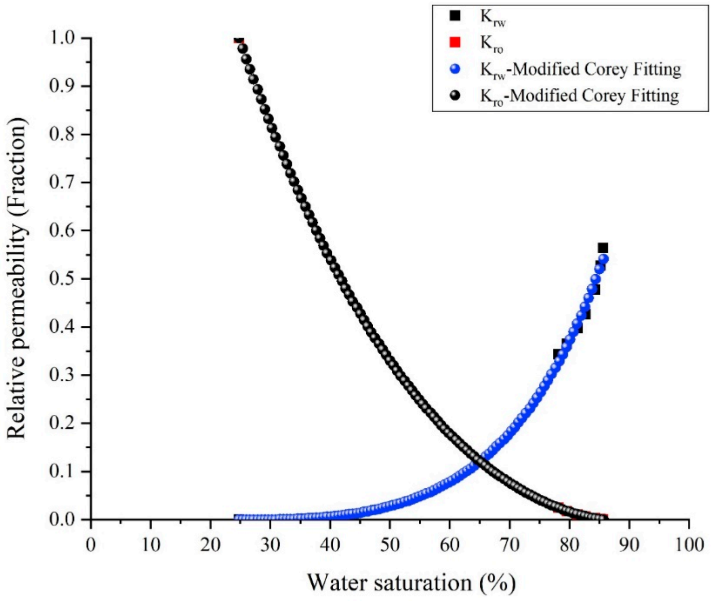

Figure S3. Water/oil relative permeability curves.

S.2 Mercury Injection Capillary Pressure (MICP) Measurements

A Micrometrics Autopore IV 9500 Porosimeter (manufactured in GA, USA) was used to precisely measure the amount of mercury being injected into the rock sample. After weighting the cleaned and dried rock sample, a proper penetrometer was selected, and the sample was placed inside the rig. Then, the assembly was loaded into the low-pressure chamber of the apparatus. By reducing the pressure within the penetrometer to less than 0.0009 psia, the penetrometer was evacuated. In this step, the bulk volume of the core cutting was estimated. The injection pressure of mercury into the rock sample increased incrementally from 0.5 to 30 psia. After equilibrium was established in the last step (i.e., 30 psia), the injection pressure lowered to ambient pressure, and the penetrometer was released. Then, the penetrometer was placed into the high-pressure chamber, mercury injection started and the pressure was raised gradually to 60000 psi. The amount of mercury entering the pores was monitored and recorded precisely at each pressure step. We reached the equilibrium point when the intrusion rate fell below 0.001 μL/g-sec. Finally, mercury saturations were calculated as a percentage of the pore volume at each pressure. The pore volume used to calculate mercury saturation was obtained from the maximum intrusion volume of mercury.

S.2.1 Procedure to Determine Pore Size Distribution by Means of MIP Experiments

During the injection of the mercury into the sample, the injected pore volume (v) versus the pore access radius (r) was plotted [6]. The differential of this value indicated the pore throat size distribution (PSD) function:

$$\mathrm{PSD} = \frac{\mathrm{dv}}{\mathrm{dlog(r)}} \quad (2)$$

The differential was calculated numerically. The central difference method was used to calculate PSD as:

$$\mathrm{PSD}_i = \frac{\mathrm{v}_{i+1} - \mathrm{v}_{i-1}}{\log(\mathrm{r}_{i+1}) - \log(\mathrm{r}_{i-1})} \quad (3)$$

PSD was smoothed as:

$$\mathrm{PSD_i} = \frac{\mathrm{PSD_{i-1}} + 2\mathrm{PSD_i} + \mathrm{PSD_{i+1}}}{4} \tag{4}$$

After that, the PSD was normalized to 1 as follows:

$$\mathrm{PSD_{normal\ i}} = \frac{\mathrm{PSD_i}}{\mathrm{PSD_{max}}} \tag{5}$$

References

1. Tóth, J.; Bodi, T.; Szűcs, P.; Civan, F. Convenient formulae for determination of relative permeability from unsteady-state fluid displacements in core plugs. *J. Pet. Sci. Eng.* **2002**, *36*, 33–44.
2. Amedi, H.R.; Ahmadi, M.A. Experimental investigation the effect of nanoparticles on the oil-water relative permeability. *Eur. Phys. J. Plus* **2016**, *131*, 125.
3. Tóth, J.; Bódi, T.; Szűcs, P.; Civan, F. Practical Method for Analysis of Immiscible Displacement in Laboratory Core Tests. *Transp. Porous Media* **1998**, *31*, 347–363.
4. Ren, X.; Li, A.; Fu, S.; Wang, S. Experimental study on the oil-water relative permeability relationship for tight sandstone considering the nonlinear seepage characteristics. *J. Pet. Sci. Eng.* **2018**, *161*, 409–416.
5. Johnson, E.; Bossler, D.; Bossler, V.N. Calculation of Relative Permeability from Displacement Experiments. *Trans. AIME* **1959**, *216*, 370–372.
6. Marle, C. *Multiphase flow in porous media.* Éditions Technip: Paris, France, 1981.

© 2020 by the authors. Licensee MDPI, Basel, Switzerland. This article is an open access article distributed under the terms and conditions of the Creative Commons Attribution (CC BY) license (http://creativecommons.org/licenses/by/4.0/).
